# Maternal mental health during COVID-19 pandemic outbreak: A cross-sectional comparative study

**DOI:** 10.3389/fpubh.2022.994004

**Published:** 2023-01-16

**Authors:** Shuliweeh Alenezi, Sahar H. Abdulghani, Lana A. Shaiba, Adnan Hadid, Rana Y. Al Ohaly, Basmah S. Aldeghaither, Rania A. Alessa, Ahmed S. Alyahya

**Affiliations:** ^1^Department of Psychiatry, College of Medicine, King Saud University, Riyadh, Saudi Arabia; ^2^SABIC Psychological Health Research and Applications Chair (SPHRAC), Department of Psychiatry, College of Medicine, King Saud University, Riyadh, Saudi Arabia; ^3^Department of Obstetrics and Gynecology, King Saud University Medical City, Riyadh, Saudi Arabia; ^4^Department of Pediatrics, College of Medicine, King Saud University Medical City, Riyadh, Saudi Arabia; ^5^Department of Neonatology, King Saud University Medical City, Riyadh, Saudi Arabia; ^6^College of Medicine, King Saud University, Riyadh, Saudi Arabia; ^7^Department of Psychiatry, Eradah Complex for Mental Health, Riyadh, Saudi Arabia

**Keywords:** mental health, maternal health, peri-partum depression, peri-partum anxiety, peri-natal, COVID-19

## Abstract

**Background:**

As COVID-19 spread in several countries, social distancing measures was implemented around the world, affecting the quality of lives for millions of people. The impact was more pronounced on vulnerable populations such as pregnant women, who are at even more risk due to their suppressed immune system. Moreover, mental health disorders are more common among pregnant women compared to non-pregnant. This study aims to assess the influence of social isolation measures due to the COVID-19 pandemic on the mental health of women in their third trimester and postpartum.

**Material and methods:**

This is a cross-sectional survey-based study conducted in Khalid University Hospital, Riyadh, Saudi Arabia, between the months of April to May 2021, to explore depression and anxiety levels in females who gave birth during the COVID-19 pandemic. In addition to background demographic data, the survey included Patient Health Questionnaire-9 (PHQ-9) and Generalized Anxiety Disorder Scale-7 (GAD-7) were utilized to detect symptoms of depression and anxiety, respectively.

**Results:**

A total of 283 women were included in this study, almost half of them were ante-natal (n-141) and the rest were post-natal (*n* = 124). 62.3% were in the age groups of 25–35 years. Based on the PHQ-9 scoring, 65% of the study sample had depression (ranging from mild to severe). Moreover, based on GAD-7 scoring, 49.1% had anxiety (ranging from mild to severe). No association was found between PHQ-9 and GAD-7 scores and different sociodemographic and obstetric factors. Additionally, the mean scores of women infected with COVID-19 vs. women who has never been diagnosed with COVID-19 were closely comparable.

**Conclusions:**

We reported a high prevalence of depression and anxiety among pregnant women during COVID-19 pandemic. Policymakers and health care providers are advised to implement targeted preventive measures for pregnant women to improve mental health in times of epidemics.

## Introduction

The coronavirus disease 2019 (COVID-19) was first identified in December 2019, in Wuhan, Hubei province, China. It was declared as a global pandemic in January 2020 and has become a novel health emergency (1, 2). Social distancing measures that have been implemented around the world during the COVID-19 pandemic have affected the quality of lives for hundreds of millions of people by changing the norms of socializing, working, studying, living together as a family and interacting with others (3). Moreover, these measures and their impact on daily life may increase the risk of developing depression among vulnerable populations, such as pregnant women (4). The impact of COVID-19 was reported in multiple vulnerable populations with elevated rates of mental health conditions, such as, pregnant women (5, 6), health care workers (7–10), university students (11, 12), elderly (13), children (14), and individuals with disability (15).

A systematic review conducted in 2019, concluded that pregnant women are vulnerable to negative psychological symptoms during natural disasters (16). Mental health disorders are common among pregnant women that manifest mainly in the form of depression or anxiety. Depression is a mood disorder that is characterized by emotional responses to certain stimuli with impaired daily functioning over a period of time; it has a variety of presentations and a broad constellation of symptoms (17, 18). Anxiety disorders are a group of mental disorders characterized by an unpleasant feeling with uneasiness, worry about future events and/or fear of responding to current events (17). Around 12% of pregnant women experience depression, and up to 22% of pregnant women experience symptoms of anxiety during late pregnancy (2). Due the use of different scales and methods of diagnosing, the prevalence of depression and anxiety varies in the existing literature. Nevertheless, in a systematic review that examined 81 studies, it was concluded that rates of depression and anxiety increased during the pandemic. Three of the included studies asked women report their mental health before, retrospectively, and during pandemic. All of them showed a general increase in levels of depression and anxiety during the pandemic (5). Moreover, one study showed that a significant increase in self-reported levels of depression and anxiety, and substantial reductions in physical activity was reported in 900 pregnant women within the first year after delivery, and this reported before and during the pandemic (19). The prevalence of reported symptoms of depression and anxiety among peri-natal women during the pandemic were 26.4–39.2% and 30.6–46.3%, respectively (1, 20–23). Furthermore, many studies have examined the association between socioeconomic factors and scores of depression and anxiety. However, the results were equivocal (24).

In addition, pregnant women are more susceptible to infection due to their naturally suppressed immune system, and they are generally considered at increased risk of severe complications (25). In addition to these two factors, worries of vertical transmission of infection to fetus contribute to increased worries in pregnant women (2). Moreover, mental health conditions can have a pronounce economic consequences when left untreated (26, 27). For example, a recently published national survey conducted in Texas, USA showed that mother-child pair with untreated maternal mental health condition costs more than 44,000 USD compared to the national average that 32,000 USD (26).

Given the above considerations, our aim in this study was to assess the influence of social isolation measures due to the COVID-19 pandemic on the mental health of women in their third trimester and postpartum. To address this aim we used a translated, standardized, and culturally appropriated scales to screen for depression and anxiety during COVID-19 Pandemic. To the best of our knowledge, this is the first study that attempts to estimate the prevalence of depression and anxiety symptoms in pregnant women in Saudi Arabia during the COVID-19 pandemic.

## Materials and methods

### Study design

This is a cross-sectional survey-based study conducted in Saudi Arabia between the months of April to May 2021, to explore depression and anxiety levels in females who gave birth during the COVID-19 pandemic.

### Sampling and participants

A convenience sample technique was utilized. Sample size was calculated using EpiInfo software version 7.2.4.0 using a large population size with an expected prevalence of depression and anxiety of 35% and margin of error of 6% based on previous research estimates (20–23). The needed sample size is at least 244 participants (122 ante-natal, and 122 post natal), using a 95% confidence level. Women were invited to participate from both outpatient clinics and delivery wards at King Khalid University Hospital, Riyadh, Saudi Arabia. The inclusion criteria were: women aged 18 years and above in their third trimester of pregnancy or immediately postpartum with no documented mental illness.

### Measures and instruments

Participants were surveyed using an Arabic questionnaire that was collected face-to-face and consisted of three sections. The first section contained questions related to background demographic data: Age, nationality, level of education, job title, and monthly income. In addition, a brief medically related questions were included: current/previous COVID-19 infection, chronic illnesses, number of pregnancies, type of pregnancy (spontaneous vs. induced), and pregnancy complications. The second section utilized an Arabic-validated assessment scales: (Patient Health Questionnaire (PHQ)-9) (28) and the Generalized Anxiety Disorder (GAD-7) scale (29). Both of these scales have been used frequently on different populations to screen for depression and anxiety, respectively, and assess their severity level (30, 31). PHQ-9 is a 9-item instrument with a 4-point likert scale. Each item is scored from 0 to 3, generating a total score ranging from 0 to 27. A total score of 0–4 indicates minimal depression, 5–9 indicates mild depression, 10–14 indicates moderate depression, 15–19 indicates moderately severe depression, and 20–27 indicates severe depression. GAD-7 is a 7-item instrument with a 4-point likert scale. Each item is scored from 0 to 3, generating a total score ranging from 0-21. A total score of 5-9 indicates mild anxiety, 10-14 indicates moderate anxiety and 15–21 indicates severe anxiety.

### Ethical consideration

#### Human accordance statement

The study was conducted according to the guidelines of the Declaration of Helsinki.

#### Study approval

Ethical approval was obtained from the Institutional Review Board at King Saud University IRB office, Riyadh, Saudi Arabia.

#### Informed consent to participate

The respondents who met the inclusion criteria and agreed to participate were aware of the study objectives and given the option to withdraw from the study at any time.

### Statistical analyses

Descriptive statistics were used in the form of raw numbers and percentages for the categorical variables. Parametric tests were used when there is normality of the distribution based on Shapiro-wilk test or the sample size is large (>30 in each group as per the central limit theorem), while non-parametric tests were used when the sample size is small with no normality of the distribution. Independent *t*-test was used to compare total scores on PHQ-9 and GAD-7 across both groups, if they have ever been diagnosed with COVID-19, presence of chronic illnesses, complications in pregnancy (maternal) and complications of pregnancy (fetal). Mann-Whitney U test was used to study the difference between total scores on PHQ-9 and GAD-7 between different nationality and types of pregnancy. One way ANOVA was used to study the difference between total scores on PHQ-9 and GAD-7 across age, level of education and number of pregnancies. Kruskal Wallis test was used to study the difference between total scores on PHQ-9 and GAD-7 across job title and monthly income. IBM SPSS 26 for windows software was used for the analysis, and a *P* < 0.05 was considered statistically significant.

## Results

Table 1 shows the sociodemographics characteristic of participants. A total of 283 women were included in this study, almost half of them were ante-natal (*n* = 141) and the other half were post-natal (*n* = 142). The highest percentage of the mothers (62.3%) were in the age groups of 25–35 years. 92.2% of the included women are Saudis, and the highest percentage of them have an undergraduate education (57.9%). 70% of them were housewives, while 23.6% were employed and 6.4% were students. Almost half of the participants (48.5%) had a monthly income of 5,000–10,000 SAR. 17.8% reported that they have not been diagnosed with COVID-19, and 25.7% reported having chronic illness. The number of pregnancies was 1–2 times in 40.1% of them, 3–5 times in 48.9% of them, and more than 5 times in 11.1%. 10 cases representing 3.6% reported having induced pregnancy. 32.5% had maternal complications while 22.4% had fetal complications.

**Table 1:** Characteristics of the study sample (*N* = 283).

		***N***	**%**
Group	Antenatal	141	49.8
	Postnatal	142	50.2
Age	< 25	33	11.7
	25–35	175	62.3
	More than 35	73	26.0
Nationality	Saudi	261	92.2
	Non-Saudi	22	7.8
Level of education	Graduate	36	12.9
	Undergraduate	162	57.9
	Illiterate, elementary or secondary	82	29.3
Job title	Student	18	6.4
	Employed	66	23.6
	Homemaker	196	70.0
Monthly income	< 5,000	54	20.3
	5,000–10,000	129	48.5
	10,000–20,000	67	25.2
	Over 20,000	16	6.0
Have you ever been diagnosed with Novel Coronavirus (COVID19)	Yes	49	17.9
	No	225	82.1
Number of pregnancies	1–2 times	105	40.1
	3–5 times	128	48.9
	More than 5 times	29	11.1
Chronic illnesses	Yes	72	25.7
	No	208	74.3
Type of pregnancy	Spontaneous	267	96.4
	Induced	10	3.6
Complications in pregnancy (Maternal)	Yes	90	32.5
	No	187	67.5
Complications of pregnant (Fetal)	Yes	62	22.4
	No	215	77.6

Table 2 displays the percentages and raw numbers of the scores on the PHQ-9 and GAD-7 scales. Based on the PHQ-9 scoring, 35% of the study sample had no depression, 44.2% had mild depression, 17.7% had moderate depression, and 3.2% had severe depression.

**Table 2:** Depression and anxiety distribution among mothers (*N* = 283).

	**Severity level**	***N***	**%**
PHQ-9 scores	No depression	99	35.0
	Mild depression	125	44.2
	Moderate depression	50	17.7
	Severe depression	9	3.2
GAD-7 scores	Minimal anxiety	141	50.9
	Mild anxiety	81	29.2
	Moderate anxiety	39	14.1
	Severe anxiety	16	5.8

Based on GAD-7 scoring, 50.9% had minimal anxiety, 29.2% had mild anxiety, 14.1% had moderate anxiety, and 5.85% had severe anxiety.

Table 3 shows the independent *t*-test used to study the difference between total scores on the PHQ-9 and GAD-7 between ante-natal and post-natal women, have not been ever diagnosed with novel coronavirus (COVID-19), chronic illnesses, complications in pregnancy (maternal) and complications of pregnancy (fetal). There is no statistically significant difference between any of the variables between the two groups.

**Table 3:** Differences of depression and anxiety levels among women across some features.

			***N***	**Mean**	**SD**	***P*-value**
Natal status	PHQ-9	Antenatal	138	6.51	4.492	0.413
		Postnatal	140	6.97	4.931	
	GAD-7	Antenatal	139	5.91	4.536	0.159
		Postnatal	138	6.72	5.089	
Having ever been diagnosed with COVID-19	PHQ-9	Yes	48	6.65	4.029	0.866
		No	222	6.77	4.930	
	GAD-7	Yes	48	6.60	5.018	0.652
		No	220	6.25	4.825	
Chronic illnesses	PHQ-9	Yes	69	6.33	4.658	0.430
		No	206	6.85	4.767	
	GAD-7	Yes	70	6.31	4.506	0.941
		No	204	6.26	4.948	
Complications in pregnancy (Maternal)	PHQ-9	Yes	88	7.09	4.580	0.379
		No	184	6.55	4.826	
	GAD-7	Yes	89	6.69	5.127	0.344
		No	182	6.09	4.677	
Complications of pregnant (Fetal)	PHQ-9	Yes	62	7.29	4.737	0.287
		No	210	6.56	4.759	
	GAD-7	Yes	61	6.93	4.560	0.244
		No	210	6.11	4.937	

One way ANOVA was used to study the differences between the total scores of PHQ-9 and GAD-7 across age, level of education and number of pregnancies (Table 4). There was no statistically significant difference for any of the variables. In order to understand our sample further, Kruskal Wallis test was used to study the difference between total scores on thr PHQ-9 and GAD-7 across job title and monthly income. There was no statistically significant difference for any of the variables (Table 4).

**Table 4:** Differences of total scores of PHQ-9 and GAD-7 across some features among women.

			***N***	**Mean**	**SD**	***P*-Value**
Age	PHQ-9	< 25	33	6.85	5.449	0.614
		25–35	172	6.52	4.572	
		More than 35	71	7.17	4.781	
	GAD-7	< 25	33	5.79	4.682	0.768
		25–35	172	6.34	4.820	
		More than 35	70	6.53	5.015	
Level of education	PHQ-9	Graduate	35	6.26	5.124	0.611
		Undergraduate	161	6.97	4.789	
		Illiterate, elementary and secondary	80	6.48	4.464	
	GAD-7	Graduate	35	5.20	4.444	0.377
		Undergraduate	161	6.43	4.943	
		Illiterate, elementary and secondary	78	6.38	4.710	
Number of pregnancies	PHQ-9	1–2 times	103	6.55	4.715	0.566
		3–5 times	126	6.94	4.932	
		More than 5 times	29	7.59	4.102	
	GAD-7	1–2 times	104	5.95	4.635	0.397
		3–5 times	125	6.77	5.142	
		More than 5 times	28	6.89	4.417	
			**Median**	**IQR**	***P*** **-Value**	
Job title	PHQ-9	Student	6	6	0.952	
		Employed	7	8		
		Homemaker	6	6		
	GAD-7	Student	6	8	0.461	
		Employed	7	8		
		Homemaker	5	6		
Monthly income	PHQ	< 5,000	6	8	0.753	
		5,000–10,000	7	8		
		10,000–20,000	6	6		
		Over 20,000	6	8		
	GAD-7	< 5,000	5	5	0.417	
		5,000–10,000	6	7		
		10,000–20,000	4	7		
		Over 20,000	6.5	8		

## Discussion

Mental health is a crucial element of patient care and general wellbeing. Women who are pregnant are especially vulnerable. Therefore, identifying risk factors and the prevalence of psychological distress will improve the quality of care. Our primary interest in this study was to examine the prevalence of symptoms indicating depression or anxiety in women in their third trimester and postpartum during COVID-19 using PHQ-9 and GAD-7. In addition, we aim to examine the factors that may affect their scores. Our findings showed that 65 percent of the pregnant ladies were affected by depression ranging in severity: 44.2 percent had mild depression, while 21.9 percent had moderate to severe depression. Compared to the literature, we reported higher numbers. It was shown that the prevalence of peri-natal women who reported symptoms of depression was 26.4–39.2% (1, 20–23). However, one study that was conducted in Iran that used PHQ-9 scale, reported a prevalence of 67.9 percent, which is comparable to our findings (32). On the other hand, 49.1 percent reported scores indicating symptoms of anxiety, 29.2 percent had mild anxiety, and 19.9 percent had moderate to severe anxiety. This goes in line with the literature; it was consistently reported that anxiety was seen in 30.6–46.3% of women in peri-natal period (1, 20–23). The variation could be explained by the different methodologies utilized by each study, and the different epidemiological stage during which the data was obtained (1). However, the prevalence of these symptoms is higher compared to pre-pandemic reports (1, 20). Additionally, a study that compared pregnant to non-pregnant women, concluded that pregnant women are significantly more depressed and more anxious (33).

Exploring the potential risk factors is necessary to help directing preventive measures when such public health crisis emerges. The literature showed contradicting results regarding associated factors; there have been no agreed conclusions regarding different sociodemographic and obstetric factors (1, 20–23, 33). Moreover, multiple studies failed to demonstrate any significant association between sociodemographic variables and increased levels of anxiety or depression (1, 33, 34). Consistently, our data did not show any significant association with age, level of education, employment status, nationality (Saudis vs. non-Saudis), monthly income, having a chronic illness, type of pregnancy, natal status (ante- vs. post-natal), and type, number, or complications of pregnancy. However, contrary to our study, some articles reported an association between higher scores of depression and anxiety with lower level of education, lower monthly income, and being nulliparous (2, 20, 21, 35). Additionally, one study found that placenta previa was associated with higher levels of depression (18). Nevertheless, others found no association with parity, level of education, lower monthly income (1, 33, 34). Furthermore, we did not find an association between being diagnosed with COVID-19 and different depression and anxiety scores. Similarly, a study that compared those who have been diagnosed with or suspected to have COVID-19, did not show difference between the two groups (34).

In conclusion, we report a high prevalence of depression and anxiety symptoms in pregnant women during the COVID-19 pandemic. We did not find any association between different variables and higher levels of depression and anxiety. However, scores on both PHQ-9 and GAD-7 were comparable across different groups. Moreover, the mean scores of women infected with COVID-19 vs. women who has never been diagnosed with COVID-19 were closely comparable. Targeted preventive measures for pregnant women to improve mental health are needed in times of natural disasters. For instance, raising awareness toward non-pharmacologic approaches that have been approved in reducing stress in pregnant women, such as mindfulness meditation, exercise, yoga, and expressive writing (36). More research is needed to examine the contributing and the protective factors for developing anxiety and depression in pregnant women.

## Limitations and future directions

Firstly, this study is subjected to the limitations of cross-sectional studies such as convenience sampling, and recall biases. Therefore causality cannot be confirmed. Secondly, the assessment tools for depression and anxiety symptoms that were used relied on self-report. Although they were validated and used as screening tools, they cannot confirm the diagnosis of any of the conditions of interest. Being a hospital-based sample from a tertiary care setting might not reflect the overall picture of pregnant women's experience in Saudi Arabia. Nevertheless, this is the first study that attempts to examine symptoms of depression and anxiety in Saudi, pregnant women during the COVID-19 pandemic. Conducting more research and including specific vulnerable patient populations such as pregnant women in pandemic and epidemic studies is important to establish a better understanding and supportive measures.

## Data availability statement

The raw data supporting the conclusions of this article will be made available by the authors, without undue reservation.

## Ethics statement

The studies involving human participants were reviewed and approved by the Institutional Review Board at the College of Medicine and King Saud University Medical City. The patients/participants provided their written informed consent to participate in this study.

## Author contributions

All authors listed have made a substantial, direct, and intellectual contribution to the work and approved it for publication.
